# Cytosolic phospholipase A2α mediates *Pseudomonas aeruginosa *LPS-induced airway constriction of CFTR -/- mice

**DOI:** 10.1186/1465-9921-11-49

**Published:** 2010-04-29

**Authors:** Yong-Zheng Wu, Mohammad Abolhassani, Mario Ollero, Fariel Dif, Naonori Uozumi, Micheline Lagranderie, Takao Shimizu, Michel Chignard, Lhousseine Touqui

**Affiliations:** 1 Unité de Défense Innée et Inflammation, Institut Pasteur, Paris, France; 2 INSERM U.874, Paris, France; 3 Laboratoire d'Immunothérapie, Institut Pasteur, Paris, France; 4 INSERM U845, Université Paris-Descartes, Paris, France; 5 Department of Biochemistry and Molecular Biology, Faculty of Medicine, The University of Tokyo, Tokyo, Japan

## Abstract

**Background:**

Lungs of cystic fibrosis (CF) patients are chronically infected with *Pseudomonas aeruginosa*. Increased airway constriction has been reported in CF patients but underplaying mechanisms have not been elucidated. *Aim*: to examine the effect of *P. aeruginosa *LPS on airway constriction in CF mice and the implication in this process of cytosolic phospholipase A2α (cPLA2α), an enzyme involved in arachidonic acid (AA) release.

**Methods:**

Mice were instilled intra-nasally with LPS. Airway constriction was assessed using barometric plethysmograph. MIP-2, prostaglandin E2 (PGE2), leukotrienes and AA concentrations were measured in BALF using standard kits and gas chromatography.

**Results:**

LPS induced enhanced airway constriction and AA release in BALF of CF compared to littermate mice. This was accompanied by increased levels of PGE2, but not those of leukotrienes. However, airway neutrophil influx and MIP-2 production remained similar in both mouse strains. The cPLA2α inhibitor arachidonyl trifluoro-methyl-ketone (ATK), but not aspirin which inhibit PGE2 synthesis, reduced LPS-induced airway constriction. LPS induced lower airway constriction and PGE2 production in cPLA2α -/- mice compared to corresponding littermates. Neither aspirin nor ATK interfered with LPS-induced airway neutrophil influx or MIP-2 production.

**Conclusions:**

CF mice develop enhanced airway constriction through a cPLA2α-dependent mechanism. Airway inflammation is dissociated from airway constriction in this model. cPLA2α may represent a suitable target for therapeutic intervention in CF. Attenuation of airway constriction by cPLA2α inhibitors may help to ameliorate the clinical status of CF patients.

## Introduction

Cystic fibrosis (CF) is the most common recessively inherited disorder in Caucasian population (1 on 2500 births) [1,2]. This disease is due to mutations in the CF transmembrane conductance regulator gene [CFTR]. The protein product of CFTR is a chloride channel expressed in epithelial cells where it regulates the luminal secretion of chloride and water transport to keep the homeostasis of mucillary clearance. Mutations of CFTR lead to dysfunction of chloride and sodium channels, and as a consequence to airway mucus dehydration and hypersecretion. This leads to airway obstruction, chronic bacterial infection by *Pseudomonas aeruginosa*, and inflammation, which result in a dramatic respiratory insufficiency. These pulmonary complications are the most leading cause of mortality in CF patients. In addition to these manifestations, increased airway constriction was reported in CF patients. Airway constriction is a common feature in CF patients that seems to be exacerbated with age, although the underlying mechanism is not known [3]. Pioneer clinical studies revealed increased levels of prostaglandins (PGs) and leukotrienes (LTs) in broncho-alveolar lavage fluids (BALF) of CF patients [4]. PGs and LTs are metabolites of arachidonic acid (AA) that is released by cytosolic phospholipase A2α (cPLA2α) [5,6]. This enzyme has been shown to play a role in various animal models of lung inflammatory diseases including induction of airway resistance in response to allergic challenge [7,8].

Taken together these findings led us to postulate that *P aeruginosa *LPS induces airway constriction in CF through an activation of AA metabolism. Since the discovery of the gene responsible for CF disease, a number of CFTR gene-targeted mouse models, such as CFTR -/- mice [9], were generated to investigate the pathophysiology of this disease. In the present study, we investigated the effect of *P. aeruginosa *LPS on airway constriction using CFTR -/- mice. Our results showed that LPS induced exacerbated airway constriction in CFTR -/- mice compared to littermate and that cPLA2α plays a key role in this process. In addition, cPLA2α induced airway constriction occurs independently from lung inflammation. The molecular mechanisms underlying airway constriction in CFTR -/- mice and their pathophysiological relevance in CF are discussed.

## Materials and methods

### Animals and reagents

CFTR-null mice (C57BL/6J Cftrm1UNC), established by gene targeting [9] were obtained from the "CDTA" UPS44 CNRS (Orleans, France). Wild type and mutant littermates were fed together by the mother until 3-4 weeks of age. CFTR-/- mice typically die shortly after weaning from intestinal obstruction. In order to increase the survival of these mice, we used a commercial osmotic laxative (Movicol^®^) which was provided continuously in the drinking water [10]. Both CFTR-/- and littermates mice received Movicol. Experiments were performed on 8-9 week-old mice.

cPLA2α-null mice were established by gene targeting as described previously [8]. Mice heterozygous for a cPLA2α mutant allele with the genetic background of the C57BL/Ola hybrid were mated. Animals were fed a standard laboratory diet and water *ad libitum*. Eight to 9 week-old mutant homozygous mice (cPLA2α -/-) and their homozygous control littermates (cPLA2α +/+) were used in this study. The protocol for animal studies were reviewed and approved by the Institute Pasteur Animal Care and Use committee in accordance with French and European guideline.

According to the experiment of Penh measurement, animals (both CFTR and cPLA2) were divided into 4 groups including saline/wild type, saline/knock-out, LPS/wild type and LPS/knock-out (n ≥ 6 for each group). Airway constriction (Penh) was monitored before and after LPS/saline instillation as detailed below. In separate groups of mice as described above (n ≥ 6 for each group), 24 h after LPS/saline treatment, cells counts in bronchoalveolar lavage fluids were determined, eicosanoid acid and cytokine were also measured. In certain experiments, total RNA was extracted from lungs of treated animals 24 h later.

### LPS and drug instillation

Mice were slightly anesthetized with ether. Anesthetized mice received intra-nasal instillation of 330 μg/kg of *P. aeruginosa *LPS (serotype 10; Sigma, St. Louis, MO) or equivalent volume of saline. In certain experiments, ATK (20 mg/kg) or aspirin (50 mg/kg) was injected intra-peritoneally 1 h before LPS challenge. The dose and route of administration of ATK and aspirin used in the present study were adopted from previous reports [11,12].

### Measurement of airway constriction

Airway constriction was assessed in conscious and freely moving mice using whole-body barometric plethysmography (Buxco Electronics, USA) according to the manufacturer's instructions and previous reports [9,13-15]. In brief, each animal was placed in a main chamber and the pressure difference between this and a reference chamber was measured with a differential pressure transducer connected to amplifier and recorded with BioSystem XA analyzer software (Buxco Electronics, Birmingham, U.K.). Airway constriction expressed as enhanced pause (Penh) was calculated as follows: Penh = (Te - Tr)/Tr(PEP/PIP), where Te is the expiratory time (seconds), Tr is the relaxation time (time of the pressure decay to 36% of total box pressure at expiration), PEP is the peak expiratory pressure (milliliters per second), and PIP is the peak inspiratory pressure (milliliters per second).

### BALF and cell counts

Twenty-four hours after LPS or saline challenge, mice were anesthetized with pentobarbital (i.p.) and the trachea was incised and cannulated. BALF were collected with saline (4 × 0.5 ml) and total cell counts were determined using a Coulter counter (Coulter-Electronics, Margency, France) as well as a Diff-Quik staining (Baxter-Dale, Dudingen, Germany) of cytospin slides for cell differential counts. Results are expressed as the number of various cell populations per ml.

### Cytokine and eicosanoid assays

PGE2, LTB4 and cysteinyl-leukotrienes (LTC4/D4/E4) concentrations were measured using enzyme immunoassay from Cayman Chemical Co (USA). The cytokine MIP2 was measured with a Kit DuoSet ELISA (R&D Systems).

### Analysis of free AA

BALF samples were extracted by a mixture of chloroform, methanol and water (4:2:1, v/v) in the presence of 15 μg of heptadecanoic acid (internal standard). After vortex and centrifugation at 800 g for 5 min, the chloroform phase was collected and dried under a nitrogen stream. Then, fatty acids were methylated and quantified by gas chromatography-mass spectrometry with the use of a gas chromatograph as previously reported [16].

### RNA extraction and quantitative PCR

Twenty-four hours after LPS/saline challenge, mice were sacrificed by intra-peritoneal injection of an overdose of pentobarbital sodium (40 mg/Kg). The chest was opened and lung perfusion with saline was performed through the pulmonary artery to remove blood. Then, the lung tissue was excised and rinsed in a lysis buffer (Qiagen, Courtaboeuf, France). After homogenization using FastPrep system (MP Biomedicals, Illkirch, France), total RNA was extracted from lung homogenate using RNeasy mini kit (Qiagen, Courtaboeuf, France) according to the manufacturer's instructions. The mRNA level was determined using an ABI 7900 Real Time PCR detection system (Applied Biosystems, Foster City, CA). In brief, the quantitative PCR was performed in 10 μl reactions that contained 1 μl of diluted cDNA, 300 nM each of forward and reverse primer, and 1× SYBR Green PCR Master Mix (Fisher scientific, Illkirch, France). Each sample was run in triplicate for each gene and relative quantity (RQ) of mRNA calculated based on the housekeeping gene HPRT. The primer sequence and PCR annealing temperature were shown in Table 1.

**Table 1:** The primer sequence and PCR annealing temperature of quantitative PCR.

gene	Forward primer	Reverse primer	Annealing Tm (°C)
COX1	5'-gcttcgtgaacataaccg-3'	5'-ggatgccagtgatagagatg-3'	58
COX2	5'-gtgcctggtctgatgatg-3'	5'-aatgcggttctgatactgg-3'	58.4
HPRT	5'-caggccagactttgttggat-3'	5'-ttgcgctcatcttaggcttt-3'	58

### Statistical analysis

Data are expressed as means ± sem of at least 6 mice in each group. Statistical analysis was performed using either unpaired Student's t test or ANOVA test for multiple groups using SPSS software and p value less than 0.05 is considered as significant.

## Results

### Enhanced airway constriction in LPS-treated CFTR -/- mice

**Figure 1:**
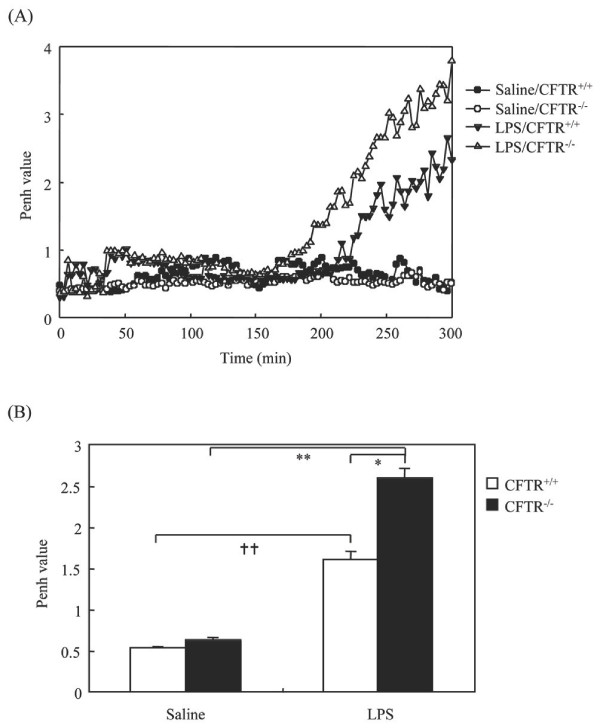
**Induction by LPS instillation of airway constriction in CFTR-/- mice**. Mice were kept in whole-body plethysmography system to measure basal level of Penh for 40 min. Then, they were subjected to intranasal instillation of either LPS (330 μg/kg) or the same volume of saline. Penh was measured continuously for 4 h and data were collected every 10 seconds and expressed as means of every 3 min. A representative graph is shown in A. The means of Penh values at the interval periods between 200 to 300 min were presented in B. * p < 0.05 LPS-treated CFTR -/- *vs *LPS-treated CFTR +/+ mice; ** p < 0.01 LPS-treated *vs *saline-treated CFTR -/- mice. †† p < 0.01 LPS-treated *vs *saline-treated CFTR +/+ mice.

We first investigated the effect of LPS (330 μg/Kg) on airway constriction in CFTR -/- and CFTR +/+ mice. This dose of LPS has been shown previously to induce an optimal airway inflammation [17]. The Penh was monitored, which reflects bronchopulmonary resistance of mice and has been described in Methods. Our results showed that untreated CFTR -/- mice exhibit similar values of Penh compared to littermate mice (Figure 1). However, instillation of LPS increased airway constriction, which occurred at higher magnitude in CFTR -/- compared to CFTR +/+ mice (Figure 1). We verified that anesthesia by itself had no effect on the measurement of Penh (data not shown).

### Airway inflammation is not different in CFTR -/- versus CFTR +/+ mice

**Figure 2:**
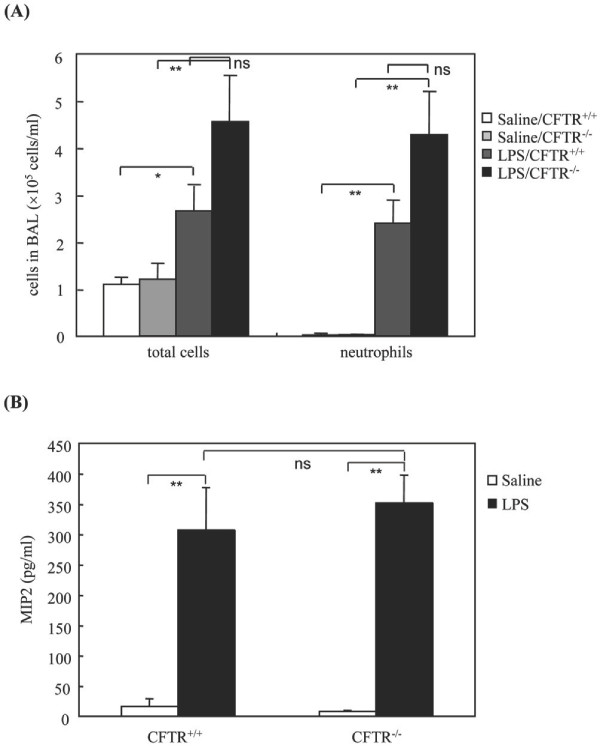
**Cell counts and MIP-2 levels in BALF**. CFTR-/- mice and their corresponding littermates were challenged either with LPS (330 μg/Kg) or saline *via *intranasal instillation. Twenty-four hours later, BALF were collected and then total cells and neutrophil counts (A) and MIP-2 levels were determined (B). * p < 0.05 LPS *vs *saline-treated CFTR+/+ mice; ** p < 0.01 LPS *vs *saline-treated mice; ns, no significant differences between LPS-treated CFTR -/- vs CFTR +/+ mice.

We next examined whether increased airway constriction is related to changes in lung inflammatory status of CFTR -/- *vs *CFTR +/+ mice. The levels of total cell and neutrophil count and MIP-2 concentrations in BALF were similar in both the mouse strains after saline challenge (Figure 2). All these parameters increased 24 h after LPS challenge but their levels remained comparable in both the mouse strains (Figure 2).

### Increased AA and PGE2 levels in BALF of CFTR -/- mice

**Figure 3:**
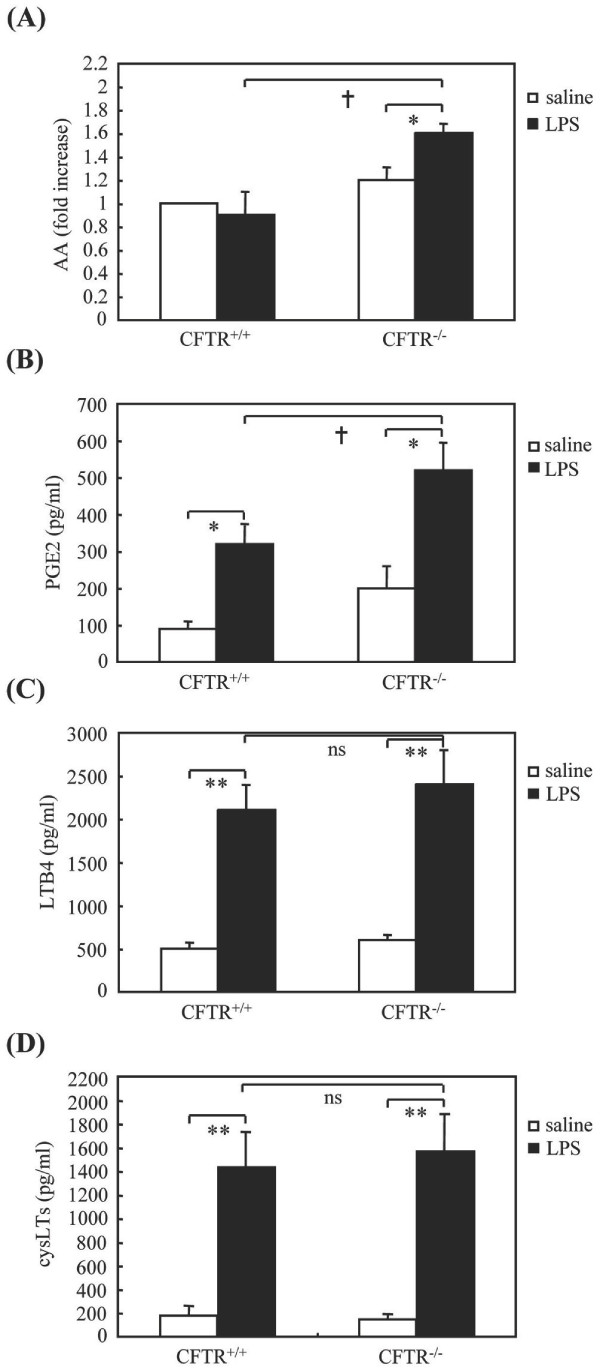
**AA, PGE2 and leukotrienes levels in BALF**. Twenty-four hours after LPS instillation, BAL were performed and levels of AA (A), PGE2 (B), LTB4 (C) and cysLTs (D) were determined. * p < 0.05 and ** p < 0.01, LPS- *vs *saline-treated mice; † p < 0.05 CFTR-/- vs CFTR +/+ mice. ns, no significant differences between LPS-treated CFTR -/- vs CFTR +/+ mice.

**Figure 4:**
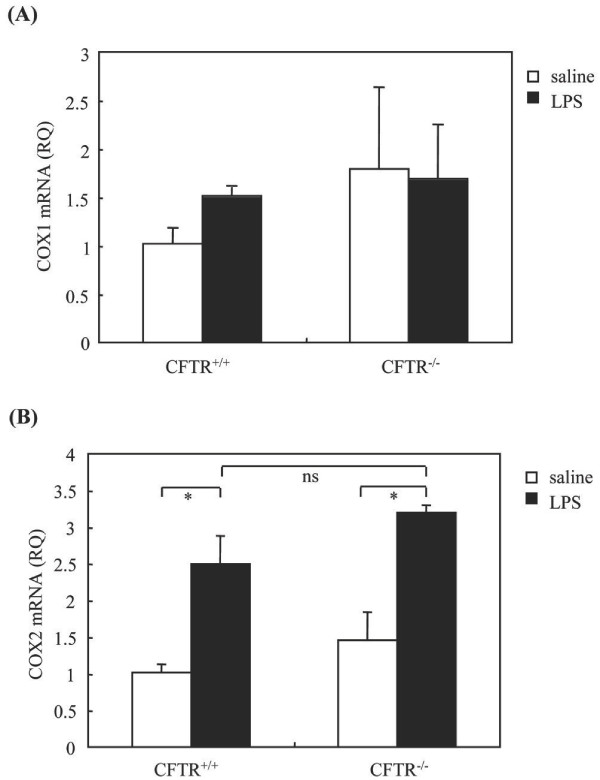
**COX mRNA levels in lung tissues after LPS stimulation**. Twenty-four hours after LPS instillation, mice were sacrificed and total RNA was extracted from homogenates of perfused lung. COX1 (A) and COX2 (B) mRNA levels were determined by quantitative PCR and expressed as RQ. RQ: relative quantity normalized to HPRT mRNA; * p < 0.05 LPS-treated *vs *saline-treated mice. ns, no significant differences between LPS-treated CFTR -/- vs CFTR +/+ mice.

Subsequent analysis revealed that CFTR -/- mice exhibited higher levels of AA in BALF as compared to CFTR +/+ mice following LPS instillation (Figure 3A). This was accompanied by an enhanced production of PGE2 (Figure 3B), whereas the levels of LTB4 (Figure 3C) and cysteinyl-leukotrienes (LTC4/D4/E4) were similar in BALF of the two mouse strains (Figure 3D). This led us to examine the pulmonary expression of COX-1 and COX-2, two major enzymes involved in PGE2 synthesis. The expression levels of COX-1 were similar in CFTR +/+ and CFTR -/- mice in basal conditions and LPS had no effect on these levels (Figure 4A). Although LPS challenge induced an increased COX-2 expression in lung tissues of CFTR-/- and CFTR +/+ mice, no significant differences were observed between these mouse strains neither before nor after LPS challenge (Figure 4B).

### A role for cPLA2α in LPS-induced airway constriction in CFTR -/- mice

**Figure 5:**
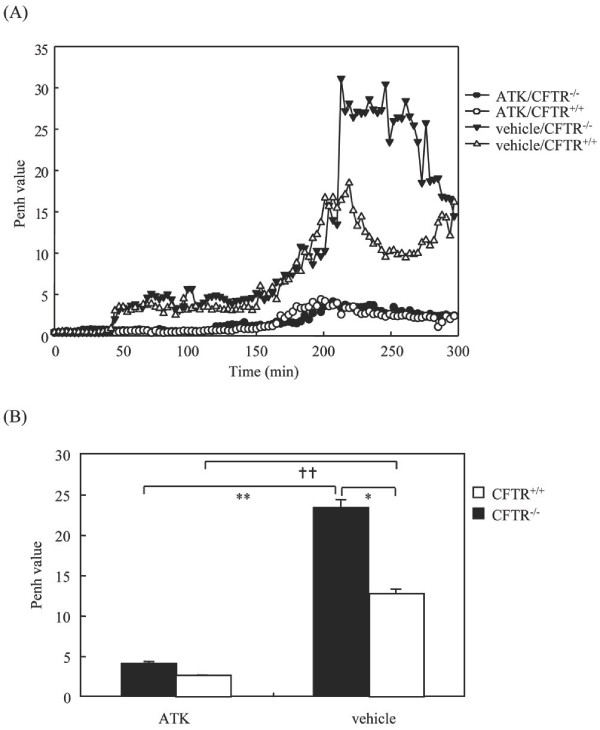
**Effect of ATK on airway constriction of CFTR-/- mice**. Either ATK (20 mg/Kg) or its vehicle, ethanol, were administered intraperitoneally to CFTR-/- and CFTR +/+ mice and basal Penh levels were measured for 40 min. Then, LPS (330 μg/kg) or saline were instilled intranasally and Penh was monitored as described before. A representative graph is shown in A. The means of Penh values at the interval periods between 200 to 300 min were presented in B. * p < 0.05 ethanol-treated CFTR -/- *vs *CFTR +/+ mice; ** p < 0.01 ethanol *vs *ATK-treated LPS challenged CFTR +/+ mice; †† p < 0.01 ethanol *vs *ATK-treated LPS challenged CFTR -/- mice.

**Figure 6:**
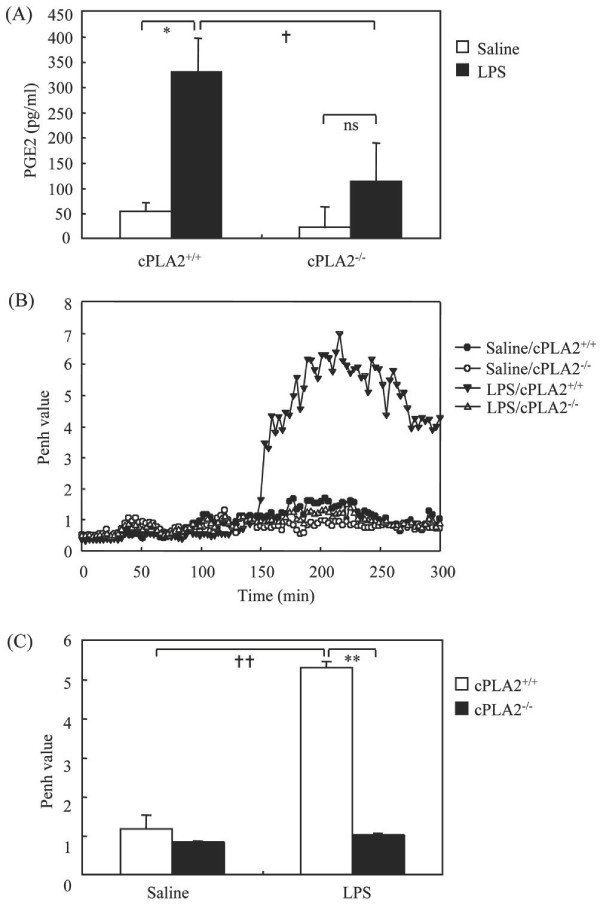
**Effect of LPS on airway constriction and PGE2 level in cPLA2α -/- mice**. PGE2 levels were determined 24 h after LPS or saline instillation (A). Basal Penh levels in both cPLA2α -/- and cPLA2α +/+ mice were measured for 40 min. Then, LPS (330 μg/kg) or saline were instilled intranasally and Penh was monitored as described before. A representative graph is shown in B. The means of Penh values at the interval times between 200 to 300 min were presented in C. * p < 0.05 and †† p < 0.01, LPS vs saline-treated mice; ** p < 0.01 and † p < 0.05, LPS-treated cPLA2α +/+ *vs *cPLA2α -/- mice; ns, no significant differences between LPS vs saline-treated CFTR -/- mice.

**Figure 7:**
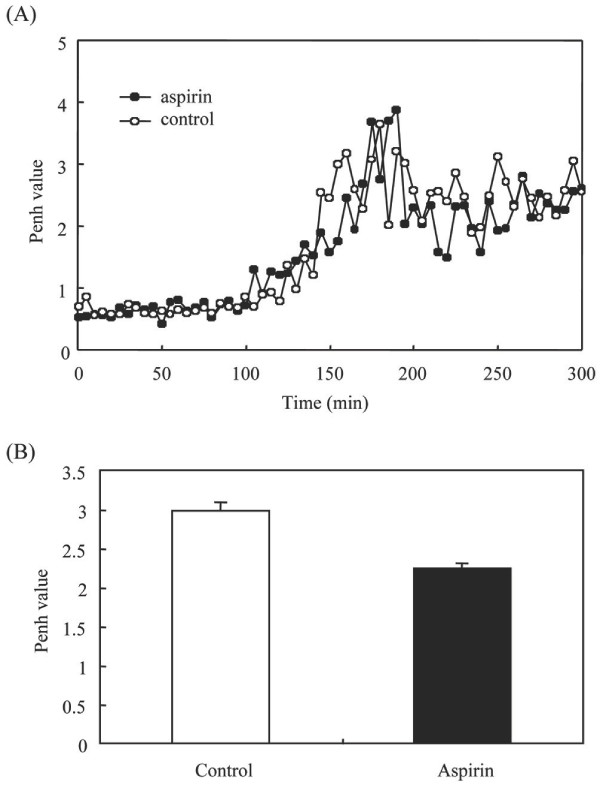
**Effect of aspirin on airway constriction of CFTR-/- mice**. Either aspirin (50 mg/Kg) or saline were injected intraperitoneally to CFTR-/- mice and basal Penh levels were measured for 40 min. Then, LPS (330 μg/kg) were introduced intranasally and Penh was monitored as described in the Methods. A representative graph is shown in A. The means of Penh values at the interval periods between 200 to 300 min were presented in B.

The findings depicted above suggested that either AA or its metabolite PGE2 may mediate enhanced airway constriction in LPS-treated CFTR -/- mice. This led us to investigate the implication of cPLA2α, the key enzyme of AA release, in LPS-induced airway constriction in CFTR -/- mice using cPLA2α inhibitor, ATK. It should be noted that in these experiments, either in ATK- or in vehicle-treated mice, the Penh values were higher to those in the other experiments (Figure 5). This is likely due to the effect of ethanol, the ATK vehicle as this solvent has been shown to exacerbate airway constriction [18,19]. In spite of ethanol effect the results showed that pretreatment of mice with ATK markedly reduced LPS-induced airway constriction compared to ethanol-treated mice (Figure 5). We next examined the effect of LPS on airway constriction using cPLA2α -/- mice. We first showed that PGE2 levels were lower in BALFs in LPS stimulated cPLA2α -/- compared to cPLA2α +/+ mice (Figure 6A), suggesting a major role of cPLA2α in PGE2 release in airways of LPS-treated mice. The figure 6B and 6C shows that LPS induced much lower airway constriction in cPLA2α -/- compared to cPLA2α +/+ mice. We next examined the role of PGE2 in LPS-induced airway constriction in CFTR -/- mice. Pretreatment of these mice with the dual COX-1/2 inhibitor aspirin had no effect on LPS-induced airway constriction (Figure 7). We verified that in our experimental conditions aspirin reduced by 90 ± 5% (mean ± sem, n = 6) PGE2 levels in BALFs of LPS-treated mice.

We also studied whether the cPLA2α/COX pathway play a role in LPS-induced neutrophil recruitment and MIP-2 production. No significant differences were observed between cPLA2α -/- and cPLA2α +/+ mice (data not shown). This was supported by the fact that instillation of ATK or aspirin had no effect on these inflammatory parameters neither before nor after LPS challenge (data not shown).

## Discussion

We report here that CFTR -/- mice develop an exacerbated airway constriction in response to LPS as compared to their corresponding littermates, which is in agreement with observations made in CF patients [3]. In addition to exacerbated pulmonary inflammation, CF patients manifest airway obstruction and wheezing [20] and near 40-50% of these patients have airway constriction. This led us to explore the mediators involved in the induction of airway constriction in CFTR -/- mice. Our studies suggest that cPLA2α, which catalyzes the key step of AA release, plays a role in enhanced airway constriction observed in CFTR -/- mice. Indeed, we found an increased concentration of AA in BALF from CFTR -/- *vs *CFTR +/+ mice. Our findings are in agreement with the pioneer work of Uozumi *et al*. reporting that cPLA2α plays a key role in increased airway resistance in response to allergic challenge [8]. Concerning the mechanism by which CFTR regulates cPLA2α, recent studies in our laboratory suggested that cPLA2α activity is inhibited by CFTR through a protein-protein interaction [[21], unpublished data (Dif F, Wu YZ)]. The absence of CFTR or its F508del mutation (known to promote CFTR degradation) may increase cPLA2α activity by the removal of the CFTR inhibitory effect.

In the present study both cPLA2α null mutation and pharmacological inhibition by the cPLA2α inhibitor, ATK, reduced LPS-induced airway constriction in CFTR -/- mice. This identifies cPLA2α as a key factor in LPS-induced airway constriction in CFTR -/- mice. However, we cannot exclude the contribution of another PLA2, iPLA2, to this process. Indeed, ATK has been shown to interfere with iPLA2 activity [22,23].

Our findings that the COX metabolites of AA did not contribute to LPS-induced airway constriction in CF animal model are in agreement with the previous studies of Vincent *et al *[24] which showed that aspirin fails to interfere with LPS-induced airway constriction in guinea pig. Aspirin did not interfere with LPS-induced airway constriction in mice and blockade of COX-2 activity by the specific inhibitor NS-398 only delayed this airway constriction [25]. In the present study we only investigated PGE2 levels since other studies have shown an increased production of various PGs including PGF1, PGF2α in BALF of LPS-treated CFTR -/- mice compared to their littermates [26]. Because aspirin is known to suppress the production of all PGs produced either by COX-1 and COX-2 pathways, we can conclude that PGs are not involved in LPS-induced airway constriction in CFTR -/- mice.

On the other hand, our findings suggest that 5-LOX, a major LOX pathway of AA metabolism is unlikely to be involved in LPS-induced airway constriction. Indeed, the levels of LTB4 and cysteinyl-leukotrienes (LTC4/D4/E4), the products of AA via LOX, are similar in CFTR-/- compared to CFTR +/+ mice. However, we cannot exclude that other LOX-dependent metabolites such as those of 12-LOX can play a role in airway constriction. Indeed, the expression level of this LOX has been shown to increase in bronchial tissues of CF patients [27].

Our studies suggest that increased PGE2 production in CFTR -/- mice may result, at least in part, from the availability of higher concentrations of free AA. This is in agreement with previous studies reporting that epithelial cells from CF patients release more AA than control cells and express higher levels of cPLA2α activity [28-30]. The fact that CFTR -/- and CFTR +/+ mice produce comparable levels of LTB4 and cysteinyl-leukotrienes is paradoxical given the enhanced production of free AA in BALF of CFTR -/- mice. Although the reasons for this paradoxical observation are still unclear, we suggest that a metabolic deviation of AA in favor of COX pathways may occur in lung tissues of CFTR -/- mice. This might be due to an enhanced activity of COX enzymes in spite of similar expression levels in lungs from CFTR -/- and CFTR +/+ mice. It is also likely that the activity of PGE synthase (PGES), which produces PGE2 from PGH2, may increase in lungs of CFTR -/- mice. Thus, in addition to changes in AA levels and cPLAa activity, an increased PGES activity could be a possible interpretation of PGE2 elevation in CFTR -/- mice.

Failure to detect changes in leukotriene levels in CFTR -/- mice is also in disagreement with the previously reported high LTB4 levels in BALF of CF patients compared to healthy subjects [4]. This discrepancy might be due to differences in the expression levels of LOX, COX and PGES in CF patients compared to CF mice. Previous findings [31] reported an exacerbated expression of COX-2 in epithelial cells and nasal polyps from CF patients as compared to the corresponding controls [31]. Whether this discrepancy is due to differences in animal species or cell types involved in COX expression remains to be investigated. It remains also unclear whether COX-2 up-regulation observed in polyps from CF patients is a direct consequence of CFTR mutation and/or a secondary consequence of airway inflammation and infection inherent to CF disease.

Although the molecular mechanisms involved in cPLA2α-induced airway constriction in LPS-challenged mice are still unclear it is likely that airway smooth muscle cells (SMC) may play a role in this process. In the asthmatic airway, acute airway constriction is caused, in part, by the enhanced presence of mediators released from inflammatory cells that directly induce bronchoconstriction and enhance bronchoconstrictor responses to other agonists. Airway obstruction and airway constriction in CF patients coincide with those seen in asthma and suggest that airway SMC remodeling may contribute to lung pathology in CF [32]. Recent studies reported that accumulation and/or hypertrophy of airway SMCs contribute to airway narrowing and airway constriction in CF patients [32,33]. A previous study showed that bradykinin-induced contraction of airway SMC occurs, in part, via a process involving a rise of [Ca^2+^] and enhanced release of AA [34]. More recently, it has been shown that the AA metabolite 20-HETE induces sustained contraction of isolated guinea pig airway SMC [35]. Interestingly, a recent study demonstrated that CFTR is also expressed in tracheal SMC and may contribute to bronchodilation [36]. Thus, it is likely that enhanced airway constriction in CFTR-/- mice might partially be due to the lack of bronchodilation function of CFTR in tracheal SMC. On the other hand, morphological analysis of the trachea and airway functional studies showed the presence of disrupted or incomplete cartilage rings in trachea of both adult and newborn CFTR -/- and F508del mice [37]. Although the loss of tracheal cartilage may predispose to collapse of the airways, the possible relationship between congenital malformations in CF mice and airway constriction remain to be investigated.

Our studies showed that although LPS induces airway constriction in CFTR-/- and cPLA2α +/+ mice at different intensity as compared to CFTR+/+ cPLA2α -/- mice, respectively, all mouse strains develop a similar extent of lung inflammation in term of neutrophil influx and MIP-2 production. This can be explained by the fact that cPLA2α may not play a role in lung inflammation in LPS challenged mice. It is also likely that PGE2 plays a role in attenuating lung inflammation in CFTR -/- mice since this prostaglandin is well known to exert an anti-inflammatory effect in lungs [38]. Thus, the enhanced production of PGE2 in CFTR -/- mice may explain, at least in part, why these mice do not exhibit exacerbated lung inflammation. The fact that airway constriction occurs independently from lung inflammation is in agreement with previous reports. Indeed, Lefort *et al*. showed that airway constriction occurs independently of pulmonary neutrophil recruitment or TNFα synthesis [39]. A similar report showed that increased airway constriction induced by inhaled LPS in COX-1 -/- and COX-2 -/- mice is dissociated from airway inflammation [15].

## Conclusions

LPS induces exacerbated airway constriction in CFTR -/- mice, which occurs through a cPLA2α-dependent mechanism and is dissociated from airway neutrophil influx and MIP-2 production. cPLA2α may represent a suitable new target for therapeutic intervention in CF.

## Abbreviations

CFTR: cystic fibrosis transmembrane conductance regulator; PGE2: prostaglandin E2; BALF: broncho-alveolar lavage fluids; AA: arachidonic acid; COX: cyclooxygenase; cPLA2α: cytosolic phospholipase A2; LTB4: leukotriene B4; ATK: arachidonyl trifluoro-methyl-ketone; Penh: enhanced pause; SMC: smooth muscle cells

## Competing interests

The authors declare that they have no competing interests.

## Authors' contributions

WYZ and LT conceived the study, planned the overall experimental design and wrote the manuscript; WYZ carried out animal instillations and analyses of inflammation; MA carried out PENH experiments, acquisition and interpretation of PENH data, MO performed measurements of arachidonic acid, FD carried out animal instillations and eicosanoid immunoassays. NU and TS produced cPLA2 KO mice and participated to manuscript writing, ML and MC participated to the conception of the project, interpretation of data and writing of the manuscript. All authors read and approved the final manuscript.
